# Relationship between Anterior Cruciate Ligament Injury and Subtalar Pronation in Female Basketball Players: Case-Control Study

**DOI:** 10.3390/jcm12247539

**Published:** 2023-12-06

**Authors:** Laura Carabasa García, Rubén Lorca-Gutiérrez, Juan Vicente-Mampel, Roser Part-Ferrer, Nadia Fernández-Ehrling, Javier Ferrer-Torregrosa

**Affiliations:** 1 Sport Podiatry Department, Catholic University of Valencia San Vicente Mártir, 46001 Valencia, Spain; laura.cg@mail.ucv.es; 2 Podiatry Department, Faculty of Medicine and Health Sciences, Catholic University of Valencia San Vicente Mártir, 46001 Valencia, Spain; ruben.lorca@ucv.es (R.L.-G.); nadia.fernandez@ucv.es (N.F.-E.); javier.ferrer@ucv.es (J.F.-T.); 3 Physiotherapy Department, Faculty of Medicine and Health Sciences, Catholic University of Valencia San Vicente Mártir, 46001 Valencia, Spain; juan.vicente@ucv.es

**Keywords:** sports injuries, subtalar pronation, ACL injury, navicular drop test, drop vertical jump test, ankle flexion, knee flexion, dynamic valgus

## Abstract

Anterior cruciate ligament (ACL) injuries are a common issue in basketball. Several studies point to subtalar pronation as a relevant risk factor for these injuries, despite their multiarticular and multiplanar nature. This study evaluated the correlation between subtalar pronation and ACL injuries in female basketball players. A total of 30 players were recruited and divided into two groups: 15 with previous ACL injury and 15 without injury. The navicular drop test (NDT) and drop vertical jump test were applied to quantify parameters such as navicular drop, calcaneal eversion, ankle dorsiflexion, knee flexion, and dynamic valgus. The results showed significantly higher NDT values (6.93 ± 1.64 mm vs. 5.41 ± 1.96 mm, *p* = 0.029) and maximum calcaneal eversion angle (10.94 ± 3.22° vs. 5.30 ± 3.33°, *p* < 0.001) in the injured group. There were also significant differences in maximum dynamic valgus (152.73 ± 15.00° vs. 165.26 ± 5.628°, *p* = 0.005) and knee flexion (93.70 ± 7.47° vs. 82.92 ± 11.14°, *p* = 0.004) between groups. These findings suggest that subtalar pronation, assessed by NDT, and calcaneal eversion could be indicators of higher susceptibility to ACL injuries in female basketball players.

## 1. Introduction

Anterior cruciate ligament (ACL) injuries have become one of the most frequent medical problems among athletes, especially in those who practice disciplines such as basketball, which require sudden changes in direction and sudden turns of the body [1,2,3]. Various epidemiological analyses agree that the incidence of ACL rupture is significantly higher in women than in men [1,3,4], probably due to anatomical, biomechanical, and hormonal factors particular to the female sex [5,6,7]. The consequences of such injuries are severe: functional limitation of the joint and increased risk of long-term osteoarthritis [8,9,10].

Most ACL tears occur without direct contact, during dynamic movements involving braking or sharp turns with the knee extended [11]. This is because the ACL acts as the main rotational stabilizer of the knee joint, preventing excessive anterior displacement of the tibia [12,13]. Its anatomical structure, attached to the femur at one end and to the tibia at the other, gives it a certain elasticity when stretched [13]. Keeping the knee extended during these dynamic movements increases the risk of ligament injury. A more extended knee during these activities carries a higher risk of injury [14]. Other associated biomechanical factors include knee hyperextension, muscle weakness [15,16,17], ligamentous laxity [18], joint asymmetries [19], and lower limb dynamic alterations. Although the classic focus has been on the sagittal plane, the multiplanar nature of these injuries is now recognized [5,20].

Several studies suggest that hyperpronation of the foot may be associated with a higher incidence of ACL injuries [18,21], as it forces the tibia into a compensatory internal rotation that is transferred to the knee joint [22]. Therefore, the present study aims to analyze the possible association between subtalar pronation and ACL injuries in female basketball players. For this purpose, clinical parameters such as the navicular descent test and the vertical jump test will be evaluated, also measuring dynamic knee valgus, knee flexion, and ankle flexion.

## 2. Materials and Methods

### 2.1. Study Design

**Figure 1:**
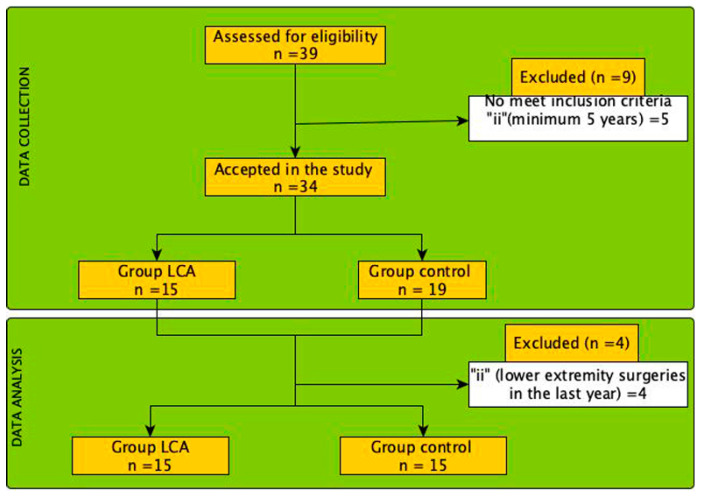
Flow diagram of the selection process and analysis of the participants included in the present study.

A descriptive–comparative study was conducted including two groups of basketball players (i.e., injured vs. non-injured basketball players) (Figure 1). This study was approved by the Research Ethics Committee of the Catholic University of Valencia (UCV/2022-2023/094) in accordance with the ethical guidelines of the Declaration of Helsinki [23]. In addition, the design and progression of participants through the trial were conducted in accordance with the STROBE guidelines [24] (see Supplementary Materials Table S1). All players were recruited from the same basketball club. Before testing procedures, all players provided written informed consent [23,24].

### 2.2. Sample Size

The sample size was calculated by taking as reference the means of 2 normal populations with equal standard deviations of 0.15. The minimum needed to reach a power of 0.95 and a bilateral α level of 0.05 with 2 groups to detect a difference of 0.2 units for a Student’s *t* test for independent samples. A total of 30 participants of fifteen subjects per group was required.

### 2.3. Participants

A total of 30 sub-elite female basketball players were selected. Factors measured are shown in Table 1. The inclusion criteria were (i) women between 14 and 28 years old, (ii) minimum 5 years of experience in professional basketball, (iii) regular training at least twice a week and 2 h per session. The exclusion criteria were (i) players with recent traumatic injuries and (ii) lower limb surgeries in the last year.

### 2.4. Assessment

#### 2.4.1. Test de Desplazamiento del Navicular (NDT)

As described by Brody [25], first, the navicular tuberosity was identified and marked on the barefoot player. Subsequently, the subject was seated, her knees were flexed to approximately 90°, and the neutral position of the subtalar joint was found. The subtalar joint was considered to be in a neutral position when the examiner, by passive inversion and eversion, could equally palpate the head of the talus on both sides with the thumb and index finger [26].

With the subject seated and the subtalar joint in a neutral position, a card was placed on the medial side of the foot that was in contact with the ground. On this card, a line was drawn to coincide with the navicular tuberosity mark. The subject was asked to relax and distribute the weight between both feet. The height of the navicular tuberosity was then re-marked on the card. The difference between the two marks was recorded in millimeters; this procedure being carried out bilaterally. NDT values greater than 7 mm [27] or 10 mm [25] were considered indicative of hi-pronation.

#### 2.4.2. Test Drop Vertical Jump (DVJ)

**Figure 2:**
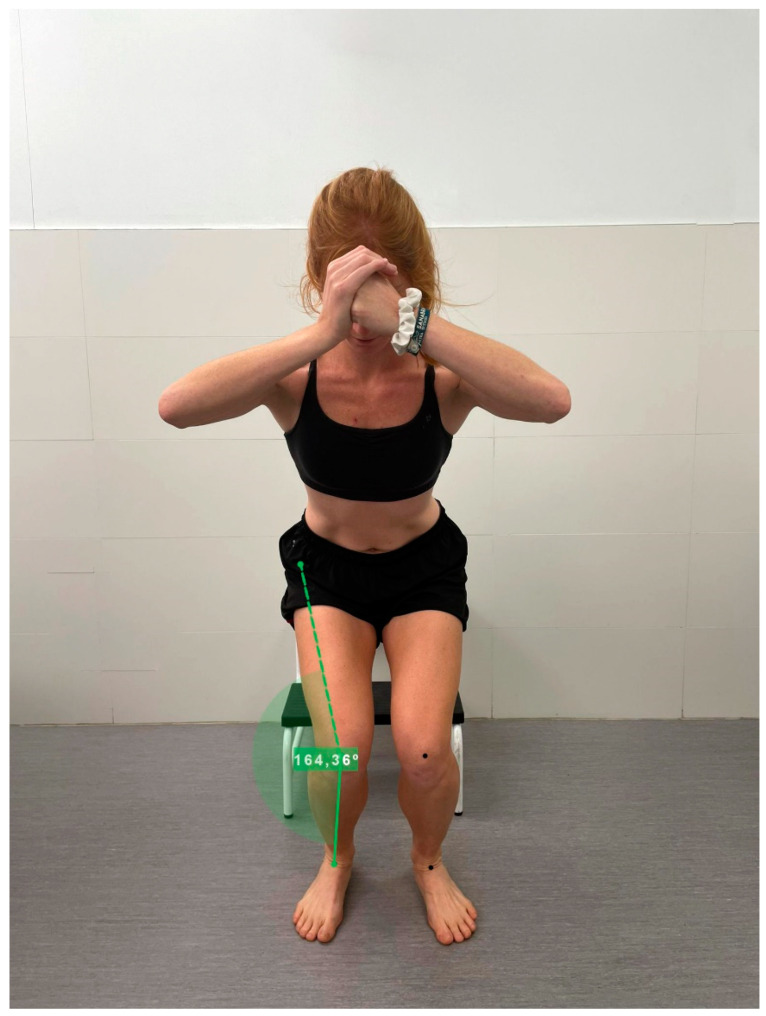
Dynamic knee valgus (DKV) after landing.

To assess the maximum dynamic valgus angle, players were placed in a common game situation, specifically during landing after a jump, a time when many injuries have been shown to occur [28]. The Drop Vertical Jump (DVJ) was used for this assessment, a tool that previous studies have shown to be useful in detecting the risk of non-contact anterior cruciate ligament (ACL) injuries [29]. In this test, the subject was asked to stand on a 31 cm high platform with feet 35 cm apart. Participants were instructed to drop straight down from the box and immediately perform a vertical jump as high as possible, raising their arms as if they were picking up a basketball rebound [30]. Each player was allowed up to 3 practice attempts (Figure 2).

If at the time of the initial landing, a higher effort than required was noted or if the participant performed a jump instead of dropping (i.e., increasing the vertical position of the center of mass when taking off from the box), the player was asked to repeat the jump [31]. Between 9 and 13 markers were placed on the players, depending on whether they were injured or not, on various anatomical structures, including the anterior superior iliac spine (EIAS), the center of the knee, and the midpoint between both malleoli in the frontal plane; greater trochanter, femoral condyle, tibial tuberosity (lateral), peroneal malleolus and head of the fifth metatarsal in the sagittal plane; and in the posterior plane, at the center of the Achilles tendon in its distal third [32].

Kinematic analysis was carried out in two dimensions (2D) to examine the alignment of the different joints, using a video camera at 240 fps (iPhone13) located two meters from the subject. The frontal plane of each participant was recorded at the time of the test.

Using the captured videos, we proceeded to measure the maximum angle of calcaneal eversion during pronation. Likewise, measurements of dynamic knee valgus, and maximum knee and ankle flexion were obtained using anatomical markers and subsequent analysis with Kinovea [33].

For all measurements, the average of the three jump attempts was taken, having practiced the exercise on 5 previous occasions.

### 2.5. Statistical Analysis

An observer who was unaware of the experimental setup performed all analyses. The mean and standard deviation (SD) were used to express the data. The Kolmogorov–Smirnov test was used to evaluate the assumption of normality. Levene’s test was also used to calculate the homogeneity of variance assumption. At *p* 0.05, the significance level was established. For the statistical analysis and graphical display of the data, SPSS 24 (SPSS 24 Inc., Chicago, IL, USA) and Jeffreys’s Amazing Statistical Package were used, respectively. To determine whether the anthropometric characteristics among the groups were homogeneous (*p* > 0.05), a one-way *t*-test was used to examine the data. The difference between injured and uninjured groups was analyzed using *t*-test for independent samples (t-Student). The ES was calculated by determining Cohen’s d coefficient, which was then expressed as the difference in standardized mean change. The ES was categorized as trivial (<0.20), small (0.20–0.59), moderate (0.60–1.19), large (1.20–1.99), or very large (>2.00) [34]. The strength of the relationship between the variables was examined using the Pearson correlation coefficient and/or Spearman correlation coefficient (for non-compliance with the normality assumption).

## 3. Results

### 3.1. Participation Flow and Sample Characteristics

A total of 30 female subjects were enrolled to participate in the study. Differences in the means of anthropometric variables across treatment assignments were analyzed using a one-way *t*-test. No evidence of heterogeneity was presented. Therefore, no analysis showed that there were no significant differences between groups (*p* > 0.05) (Table 1).

### 3.2. Results Subtalar Pronation Outcomes

Subtalar pronation outcomes were taken at the specified two groups: injured and uninjured participants. These measurements are detailed in Table 2.

**Figure 3:**
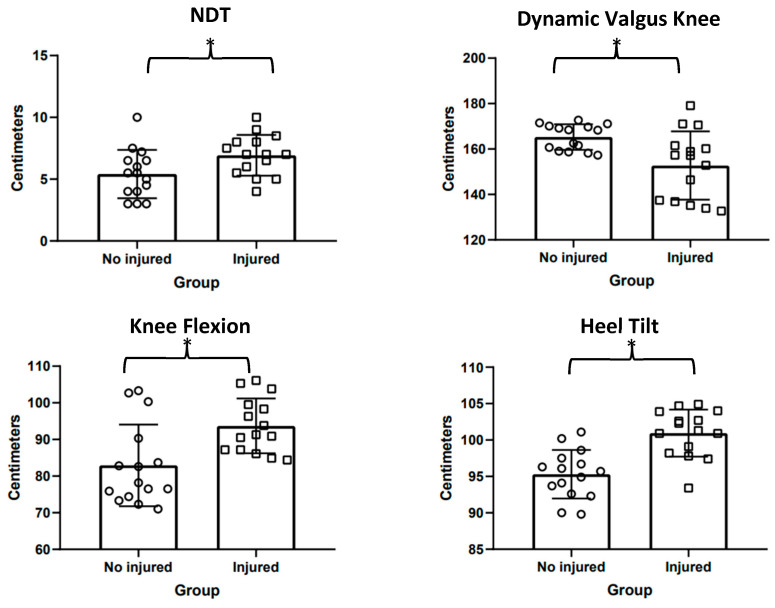
The figure shows the differences produced on main subtalar pronation outcomes by group (i.e., Uninjured and Injured). * Significant differences between groups *p* value > 0.05.

The *t*-test for independent samples showed statistically significant differences (*p* = 0.029) on NDT evaluation. Between groups (mean difference [CI 95%, t_(df)_, ES]) was in favor of the injured group (1.52 [0.16–2.87, 2.30_(28)_, 0.84]) in female basketball players. The effect size was categorized as moderate (0.08–1.58). The dynamic knee valgus *t*-test for independent samples showed statistically significant differences (*p* = 0.005). Between groups (mean difference [CI 95%, t_(df)_, ES] was in favor of the uninjured group (−12.53 [−21.00–−4.05, −3.02_(28)_, 0.84]) in female basketball players. The effect size was categorized as moderate (−1.86 to −0.32). Related to knee flexion, the analysis showed that statistically significant differences (*p* = 0.004) between group (mean difference [CI 95%, t_(df)_, ES] high knee flexion values in favor of the injured group were found (10.78 [3.46–3.69, 3.11(28), 1.13]). The effect size was categorized as moderate (0.35–1.90). Finally, the *t*-test for independent samples showed statistically significant differences (*p* = 0.001) in heel tilt. Between-group (mean difference [CI 95%, t_(df)_, ES] high tilt values in favor of the injured group were found (5.63 [3.17–8.08, 3.11(28), 1.71]) (see Figure 3). The effect size was categorized as large (0.86–2.54).

### 3.3. Relationships between Subtalar Pronation Outcomes and Injured ACL Participants

**Figure 4:**
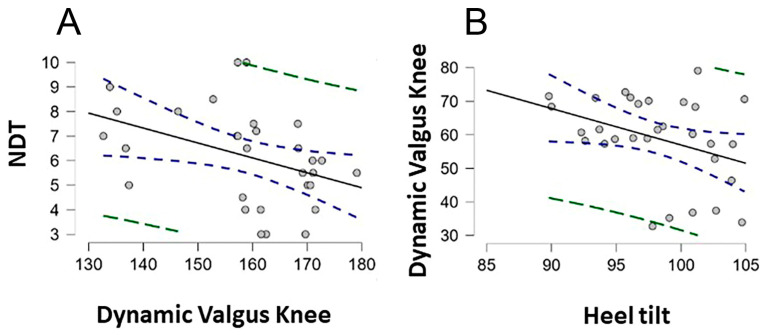
The graph illustrates the scatter plot between the dependent variables analyzed, (**A**) NDT—Dynamic Valgus Knee, (**B**) Dynamic Valgus Knee—Heel Tilt. Notes. Green marks show Prediction Intervals (95%). Blue marks show confidence intervals (95%).

The results of Spearman correlations were used to examine the relationships between clinical subtalar pronation within both groups (Figure 4). NDT was associated with dynamic knee valgus (rho = −0.513, *p* = 0.004) and heel tilt (rho = −0.375, *p* = 0.041). There was no significant relationship between the rest subtalar pronation outcomes. Complete estimates from the correlation analysis are shown in Table 3.

### 3.4. Adverse Events

During the follow-up period, no adverse events or unintended effects were recorded.

## 4. Discussion

This study aimed to analyze the possible association between subtalar pronation outcomes and ACL injury in female basketball players, by comparing a group with a previous ACL injury and a control group. The hypothesis posited that higher value subtalar pronation outcomes would be observed after the injury ACL. The primary finding of this study was in the values of the navicular drop test and the maximum angle of calcaneal tilt during the dynamic landing phase, both being higher in players with ACL injury. In our study, the participant sample consisted exclusively of women. Indeed, there are noted gender differences among the ACL injured. Female athletes are substantially more susceptible than males to suffer acute non-contact anterior cruciate ligament injury. Moreover, previous research has shown that with an uninjured extremity, females may have larger hip flexion angles, smaller hip adduction moments, larger anterior knee joint forces, larger knee extension moments, and smaller ankle inversion angles as compared to males after ACL reconstruction [35]. These results are consistent with some previous studies suggesting that hyperpronation may increase the risk of ACL injury [36] as it generates a compensatory internal tibial rotation that moves proximally to the knee joint, and other studies comparing ACL-injured and non-injured athletes have found excessive navicular drop and subtalar pronation to be more frequent risk factors for injury [37].This propagates rotational strains proximally to the knee joint. With the knee near extension during maneuvers like landing, the ACL lacks active muscular contributions to share the load [38]. Thus, with the tibia internally rotated from pronation below, the ACL can rupture if overloaded beyond capacity. A statistically significant difference was found.

Other authors, however, have not found such an association, and thus the evidence is conflicting [39] A limitation in this area is the lack of clearly established standard values for pronation parameters, where NDT values greater than 7 mm [27] or 10 mm [25] would be considered hyperpronation. However, with dynamic calcaneal eversion, there are no articles that determine normal values. Authors who have studied calcaneal eversion have obtained normal values in static. Some of them agree that it should be between 4 and 8° [40,41], while others argue that a calcaneal eversion of >5° should be considered abnormal [42]. We are therefore faced with a lack of evidence between the cause and effect of hyperpronation and ACL injuries [43].

Anterior cruciate ligament (ACL) injury is one of the most common injuries in female basketball players. The main non-contact mechanisms occur in 70% to 84% of injuries [44,45] during landings after vertical jumps, usually due to inadequate absorption of ground reaction forces as the knee is almost in full extension. Actually, in this research, 14 of the 15 injured players (*n* = 93%) were injured without contact with another element and only 1 of them (*n* = 7%) had contact at the time of injury.

As described above, the ACL is responsible for controlling the anterior motion of the tibia and inhibiting extreme ranges of tibial rotation [46]. These events occur primarily in the knee, which primarily moves on the sagittal plane, and there are authors who have studied the relationship of knee flexion with the ACL. Some have concluded that the more the joints flex during landing, the more energy is absorbed and the less the impact is transmitted to the knee, decreasing the tensile forces on the ligament [14]. Even Gabriel et al. [47] calculated at which degrees of flexion the maximum forces on the AM and PL fascicle of the ACL were found; 0° of flexion for the PL fascicle and 60° and 90° of flexion for the AM fascicle.

The difference in knee flexion between injured and uninjured players in this study was statistically significant (*p* < 0.05) (Table 1). A lower maximum knee flexion angle indicates a higher knee flexion capacity and consequently better shock absorption. The average maximum knee flexion angle of the uninjured players is 82.9°, whereas for the injured players, it is 93.7°. Thus, it is observed that the uninjured players have a greater knee flexion, with a difference of 10.8°.

Taking into consideration the study by Gabriel et al. [47], the averaged maximum knee flexion angle of the injured players (93.7°) is very close to the position in which the AM fascicle of the ACL receives the highest stress (90°).

Additionally, players with ACL injury in this study had a significantly higher average maximum dynamic knee valgus angle (152.73° vs. 165.27°), as well as a lower maximum knee flexion during the drop and jump action measured. This pattern increases the straining forces on the ACL, and therefore could also contribute to the increased risk of injury.

Excessive pronation, combined with sporting activities involving jumping and sudden changes in direction, may increase load and stress on the patellar tendon, potentially contributing to the development of knee pathology [48]. The relationship between the Q-angle and pronation is relevant, as an elevated Q-angle may predispose to excessive pronation, thereby increasing stress on the patellar tendon and contributing to the risk of injury in athletes participating in sports that require repetitive and sudden movements [49]. This biomechanical understanding may be crucial in identifying and addressing risk factors, as well as in designing more effective prevention and rehabilitation strategies for athletes with this type of injury [50]. The biomechanical understanding of the risk factors may be crucial to identify and address risk factors, as well as to design more effective prevention and rehabilitation strategies for athletes with these types of injuries.

The average age of the players at the time of injury was 17.8 years, which is consistent with previous reports suggesting an increased risk in adolescents, as already established by studies [51], probably due to high physical activity, accelerated bone growth, and hormonal changes during puberty [52]. However, in this sample, a history of previous ACL injury was not a primary factor.

In conclusion, in this cohort of female basketball players, subtalar hyperpronation assessed by static and dynamic clinical parameters was related to a history of ACL injury. While further research is required, this biomechanical factor could be modifiable and potentially used to develop specific preventive strategies [53]. These injury prevention programs should focus on controlling excessive pronation through exercises that strengthen the intrinsic musculature of the foot and improve dynamic alignment. Trainers could incorporate such exercises into warm-ups. Orthotic insoles or motion-controlled shoes may also help to optimize foot biomechanics, although studies suggest otherwise for navicular drops of less than 8 mm [54]. Likewise, training landing technique after jumps, with an emphasis on soft landings with increased knee/hip flexion, would optimize shock absorption and reduce ACL stress. Feedback to avoid dynamic knee valgus during jumps and turns could also reduce the risk of injury. Pre-participatory assessment of navicular drop and lower limb alignment during landings could identify athletes requiring interventions to correct risk factors such as hyperpronation. Finally, after ACL reconstruction, addressing pronation and muscle imbalances through the kinetic chain could optimize dynamic stability and prevent re-injury.

### Limitations and New Lines of Research

The present study has some limitations that should be considered: a small sample size (30 participants in total) reduces statistical power and makes it difficult to extrapolate results; although, the sample calculation was adequate, a larger sample would give greater support to the conclusions. Furthermore, the assessment of pronation was based only on clinical tests (NDT and calcaneal eversion), so incorporating more objective biomechanical analyses (gait platforms, opto-electronic systems) would have provided a more accurate quantification. Also, it is recommended to conduct longitudinal studies in basketball teams, examining more biomechanical parameters and performing comprehensive assessments of ligamentous-mental laxity and muscle strength, to compare variables before and after injury and establish causal relationships.

This line of research will allow studies to be carried out to evaluate the efficacy of clinical interventions in the control of excessive pronation and their impact on the prevention of ACL injuries. Specifically, randomized trials could be designed to compare the incidence of such injuries in athletes using insoles, muscle-strengthening programs, or proprioceptive training, vs. a control group. If their protective effect against injury is confirmed, these therapeutic strategies could be incorporated into prevention programs in at-risk populations.

Variable degrees of pronation could also be modeled by finite element studies and computational analysis. These biomechanical models would allow estimation of the resulting forces on the ACL at different angles of subtalar pronation. The results would help to establish optimal ranges and pronation limits to minimize the load on the anterior cruciate ligament during sports practice.

## 5. Conclusions

In conclusion, the present study demonstrated that subtalar hyperpronation, measured by the navicular drop test and calcaneal eversion, was associated with an increased risk of ACL injury among the female basketball players tested. Players with a previous ACL injury showed greater dynamic knee valgus compared to the control group, which could also increase stress on the ligament. A significant reduction was observed in the maximum knee flexion angle during the dynamic maneuvers, which implies a reduced ability to absorb forces. Moreover, no significant differences were found in maximum ankle flexion between the injured and uninjured groups. It is concluded that subtalar hyperpronation appears to be a modifiable biomechanical factor associated with an increased risk of ACL injury in young female basketball players.

## Figures and Tables

**Table 1:** Descriptions and *t*-tests anthropometric features sample by group. Data were expressed as mean and standard deviation (SD).

Outcome	All Participants (*n* = 30)	Injured (*n* = 15)	Uninjured (*n* = 15)	*p*-Value
Age	23.067 ± 4.24	23.80 ± 3.97	22.33 ± 4.51	0.353
Height cm	167.77 ± 5.23	167.33 ± 5.91	168.20 ± 4.61	0.658
Weight Kg	61.7 ± 8.74	62.53 ± 9.00	60.87 ± 8.70	0.610
Body mass index, kg/m^2^	21.87 ± 2.41	22.29 ± 2.49	21.45 ± 2.32	0.348

**Table 2:** Descriptions and *t*-tests subtalar pronation outcomes sample per group. Data were expressed as mean and standard deviation (SD).

Outcome	All Participants (*n* = 30)	Injured (*n* = 15)	Uninjured (*n* = 15)
NDT	6.173 ± 1.94	6.93 ± 1.64	5.41 ± 1.96
Dynamic Knee Valgus	159.00 ± 12.83	152.73 ± 15.00	165.26 ± 5.628
Knee Flexion	88.31 ± 10.81	93.70 ± 7.47	82.92 ± 11.14
Dorsiflexion Ankle	113.05 ± 7.07	113.33 ± 7.69	112.76 ± 6.67
Heel Tilt	8.12 ± 4.31	10.94 ± 3.22	5.30 ± 3.33

**Table 3:** Estimates from the correlation analysis (Spearman). Data were expressed as Rho and *p* value.

Outcome	Dynamic Knee Valgus	Knee Flexion	Dorsiflexion Ankle	Heel Tilt
NDT	−0.513; 0.004 **	0.142; 0.454	0.213; 0.259	0.268; 0.152
Dynamic Knee Valgus	-	−0.299; 0.109	0.145; 0.446	−0.375; 0.041 **
Knee Flexion	0.142; 0.454	-	0.254; 0.176	0.136; 0.473
Dorsiflexion Ankle	0.213; 0.259	0.254; 0.176	-	−0.122; 0.522
Heel Tilt	0.268; 0.152	0.136; 0.437	−1.122; 0.522	-

Rho = Value Spearman correlation between two variables. ** *p*-value less than 0.05 is typically considered to be statistically significant.

## Data Availability

The data presented in this study are available upon request to the corresponding author.

## Supplementary Materials

The following supporting information can be downloaded at: https://www.mdpi.com/article/10.3390/jcm12247539/s1. Table S1. STROBE Statement—Checklist of items that should be included in reports of cross-sectional studies.

## References

[1] Bram J.T., Magee L.C., Mehta N.N., Patel N.M., Ganley T.J. (2020). Anterior Cruciate Ligament Injury Incidence in Adolescent Athletes: A Systematic Review and Meta-Analysis. Am. J. Sport. Med..

[2] Takahashi S., Nagano Y., Ito W., Kido Y., Okuwaki T. (2019). A Retrospective Study of Mechanisms of Anterior Cruciate Ligament Injuries in High School Basketball, Handball, Judo, Soccer, and Volleyball. Medicine.

[3] Beynnon B.D., Vacek P.M., Newell M.K., Tourville T.W., Smith H.C., Shultz S.J., Slauterbeck J.R., Johnson R.J. (2014). The Effects of Level of Competition, Sport, and Sex on the Incidence of First-Time Noncontact Anterior Cruciate Ligament Injury. Am. J. Sport. Med..

[4] McCarthy M.M., Voos J.E., Nguyen J.T., Callahan L., Hannafin J.A. (2013). Injury Profile in Elite Female Basketball Athletes at the Women’s National Basketball Association Combine. Am. J. Sport. Med..

[5] Quatman C.E., Quatman-Yates C.C., Hewett T.E. (2010). A “plane” Explanation of Anterior Cruciate Ligament Injury Mechanisms: A Systematic Review. Sport. Med..

[6] Raymond-Pope C.J., Dengel D.R., Fitzgerald J.S., Nelson B.J., Bosch T.A. (2020). Correction: Anterior Cruciate Ligament Reconstructed Female Athletes Exhibit Relative Muscle Dysfunction after Return to Sport. Int. J. Sport. Med..

[7] Larwa J., Stoy C., Chafetz R.S., Boniello M., Franklin C. (2021). Stiff Landings, Core Stability, and Dynamic Knee Valgus: A Systematic Review on Documented Anterior Cruciate Ligament Ruptures in Male and Female Athletes. Int. J. Environ. Res. Public Health.

[8] Webster K.E., Hewett T.E. (2022). Anterior Cruciate Ligament Injury and Knee Osteoarthritis: An Umbrella Systematic Review and Meta-Analysis. Clin. J. Sport Med..

[9] Ramos-Mucci L., Elsheikh A., Keenan C., Eliasy A., D’Aout K., Bou-Gharios G., Comerford E., Poulet B. (2022). The Anterior Cruciate Ligament in Murine Post-Traumatic Osteoarthritis: Markers and Mechanics. Arthritis Res. Ther..

[10] Rajput V., Haddad F.S. (2022). Is the Die Cast? Anterior Cruciate Ligament Injury and Osteoarthritis. Bone Joint J..

[11] Zou J., Yang W., Cui W., Li C., Ma C., Ji X., Hong J., Qu Z., Chen J., Liu A. (2023). Therapeutic Potential and Mechanisms of Mesenchymal Stem Cell-Derived Exosomes as Bioactive Materials in Tendon-Bone Healing. J. Nanobiotechnol..

[12] Hassebrock J.D., Gulbrandsen M.T., Asprey W.L., Makovicka J.L., Chhabra A. (2020). Knee Ligament Anatomy and Biomechanics. Sports Med. Arthrosc. Rev..

[13] Zhang Q., Adam N.C., Hosseini Nasab S.H., Taylor W.R., Smith C.R. (2021). Techniques for In Vivo Measurement of Ligament and Tendon Strain: A Review. Ann. Biomed. Eng..

[14] Yu B., Lin C.-F., Garrett W.E. (2006). Lower Extremity Biomechanics during the Landing of a Stop-Jump Task. Clin. Biomech..

[15] Ghanati H.A., Letafatkar A., Shojaedin S., Hadadnezhad M., Schöllhorn W.I. (2022). Comparing the Effects of Differential Learning, Self-Controlled Feedback, and External Focus of Attention Training on Biomechanical Risk Factors of Anterior Cruciate Ligament (ACL) in Athletes: A Randomized Controlled Trial. Int. J. Environ. Res. Public Health.

[16] Ferri-Caruana A., Prades-Insa B., Serra-AÑÓ P. (2020). Effects of Pelvic and Core Strength Training on Biomechanical Risk Factors for Anterior Cruciate Ligament Injuries. J. Sports Med. Phys. Fitness.

[17] Jeong J., Choi D.H., Shin C.S. (2021). Core Strength Training Can Alter Neuromuscular and Biomechanical Risk Factors for Anterior Cruciate Ligament Injury. Am. J. Sport. Med..

[18] Alahmri F., Alsaadi S., Ahsan M., Alqhtani S. (2022). Determining the Knee Joint Laxity between the Pronated Foot and Normal Arched Foot in Adult Participants. Acta Biomed..

[19] Söderman K., Alfredson H., Pietilä T., Werner S. (2001). Risk Factors for Leg Injuries in Female Soccer Players: A Prospective Investigation during One out-Door Season. Knee Surg. Sport. Traumatol. Arthrosc..

[20] Shimokochi Y., Shultz S.J. (2008). Mechanisms of Noncontact Anterior Cruciate Ligament Injury. J. Athl. Train..

[21] Mafi M., Sheikhalizade H., Jafarnezhadgero A.A., Asheghan M. (2023). Investigating the Effect of Sand Training on Running Mechanics in Individuals with Anterior Cruciate Ligament Reconstruction and Pronated Feet. Gait Posture.

[22] Hodel S., Torrez C., Flury A., Fritz B., Steinwachs M.R., Vlachopoulos L., Fucentese S.F. (2022). Tibial Internal Rotation in Combined Anterior Cruciate Ligament and High-Grade Anterolateral Ligament Injury and Its Influence on ACL Length. BMC Musculoskelet. Disord..

[23] Association W.M. (2013). World Medical Association Declaration of Helsinki: Ethical Principles for Medical Research Involving Human Subjects. JAMA.

[24] Vandenbroucke J.P., Von Elm E., Altman D.G., Gøtzsche P.C., Mulrow C.D., Pocock S.J., Poole C., Schlesselman J.J., Egger M. (2007). Strengthening the Reporting of Observational Studies in Epidemiology (STROBE): Explanation and Elaboration. PLoS Med..

[25] Brody D.M. (1982). Techniques in the Evaluation and Treatment of the Injured Runner. Orthop. Clin. North Am..

[26] Beckett M.E., Massie D.L., Bowers K.D., Stoll D.A. (1992). Incidence of Hyperpronation in the ACL Injured Knee: A Clinical Perspective. J. Athl. Train..

[27] Gould N. (1983). Evaluation of Hyperpronation and Pes Planus in Adults. Clin. Orthop. Relat. Res..

[28] Hutchinson M.R., Ireland M.L. (1995). Knee Injuries in Female Athletes. Sports Med..

[29] Hewett T.E., Myer G.D., Ford K.R., Heidt R.S., Colosimo A.J., McLean S.G., Van Den Bogert A.J., Paterno M.V., Succop P. (2005). Biomechanical Measures of Neuromuscular Control and Valgus Loading of the Knee Predict Anterior Cruciate Ligament Injury Risk in Female Athletes: A Prospective Study. Am. J. Sport. Med..

[30] Ford K.R., Myer G.D., Hewett T.E. (2003). Valgus Knee Motion during Landing in High School Female and Male Basketball Players. Med. Sci. Sports Exerc..

[31] Krosshaug T., Steffen K., Kristianslund E., Nilstad A., Mok K.M., Myklebust G., Andersen T.E., Holme I., Engebretsen L., Bahr R. (2016). The Vertical Drop Jump Is a Poor Screening Test for ACL Injuries in Female Elite Soccer and Handball Players. Am. J. Sport. Med..

[32] Herrington L. (2011). Knee Valgus Angle during Landing Tasks in Female Volleyball and Basketball Players. J. Strength Cond. Res..

[33] Puig-Diví A., Escalona-Marfil C., Padullés-Riu J.M., Busquets A., Padullés-Chando X., Marcos-Ruiz D. (2019). Validity and Reliability of the Kinovea Program in Obtaining Angles and Distances Using Coordinates in 4 Perspectives. PLoS ONE.

[34] Hopkins W.G., Marshall S.W., Batterham A.M., Hanin J. (2009). Progressive Statistics for Studies in Sports Medicine and Exercise Science. Med. Sci. Sports Exerc..

[35] Vij N., Tummala S., Vaughn J., Chhabra A., Salehi H., Winters J., Browne A., Glattke K., Brinkman J.C., Menzer H. (2023). Biomechanical Gender Differences in the Uninjured Extremity after Anterior Cruciate Ligament Reconstruction in Adolescent Athletes: A Retrospective Motion Analysis Study. Cureus.

[36] Nilstad A., Andersen T.E., Bahr R., Holme I., Steffen K. (2014). Risk Factors for Lower Extremity Injuries in Elite Female Soccer Players. Am. J. Sport. Med..

[37] Allen M.K., Glasoe W.M. (2000). Metrecom Measurement of Navicular Drop in Subjects with Anterior Cruciate Ligament Injury. J. Athl. Train..

[38] Rabe K.G., Segal N.A., Waheed S., Anderson D.D. (2018). The Effect of Arch Drop on Tibial Rotation and Tibiofemoral Contact Stress in Postpartum Women. PM&R.

[39] Hetsroni I., Finestone A., Milgrom C., Ben Sira D., Nyska M., Radeva-Petrova D., Ayalon M. (2006). A Prospective Biomechanical Study of the Association between Foot Pronation and the Incidence of Anterior Knee Pain among Military Recruits. J. Bone Jt. Surg. -Ser. B.

[40] Woodford-Rogers B., Cyphert L., Denegar C.R. (1994). Risk Factors for Anterior Cruciate Ligament Injury in High School and College Athletes. J. Athl. Train..

[41] Åström M., Arvidson T. (1995). Alignment and Joint Motion in the Normal Foot. J. Orthop. Sport. Phys. Ther..

[42] LeLiévre J. (1970). Current Concepts and Correction in the Valgus Foot. Clin. Orthop. Relat. Res..

[43] Myers C.A., Hawkins D. (2010). Alterations to Movement Mechanics Can Greatly Reduce Anterior Cruciate Ligament Loading without Reducing Performance. J. Biomech..

[44] Faunø P., Jakobsen B.W. (2006). Mechanism of Anterior Cruciate Ligament Injuries in Soccer. Int. J. Sports Med..

[45] Boden B.P., Dean C.S., Feagin J.A., Garrett W.E. (2000). Mechanisms of Anterior Cruciate Ligament Injury. Orthopedics.

[46] Siegel L., Vandenakker-Albanese C., Siegel D. (2012). Anterior Cruciate Ligament Injuries: Anatomy, Physiology, Biomechanics, and Management. Clin. J. Sport Med..

[47] Gabriel M.T., Wong E.K., Woo S.L.-Y., Yagi M., Debski R.E. (2004). Distribution of in Situ Forces in the Anterior Cruciate Ligament in Response to Rotatory Loads. J. Orthop. Res..

[48] Kızılgöz V., Sivrioğlu A.K., Ulusoy G.R., Aydın H., Karayol S.S., Menderes U. (2018). Analysis of the Risk Factors for Anterior Cruciate Ligament Injury: An Investigation of Structural Tendencies. Clin. Imaging.

[49] Todor A., Stetson W.B., Felu´s J.F., Genç A.S., Güzel N. (2023). Patellofemoral Angle, Pelvis Diameter, Foot Posture Index, and Single Leg Hop in Post-Operative ACL Reconstruction. Medicina.

[50] Aiyegbusi A., Tella B., Okeke C. (2019). Variáveis Biomecânicas Dos Membros Inferiores São Indicadores Do Padrão de Apresentação Da Tendinopatia Patelar Em Atletas de Elite Africanos de Basquetebol e Voleibol. Rev. Bras. Ortop..

[51] Tovar-Cuellar W., Galván-Villamarín F., Ortiz-Morales J. (2018). Complications Associated with the Techniques for Anterior Cruciate Ligament Reconstruction in Patients under 18 Years Old: A Systematic Review. Rev. Española De Cirugía Ortopédica Y Traumatol. (Engl. Ed.).

[52] Hewett T.E., Myer G.D., Ford K.R. (2004). Decrease in Neuromuscular Control About the Knee with Maturation in Female Athletes. J. Bone Jt. Surg..

[53] Hewett T.E., Myer G.D., Ford K.R., Paterno M.V., Quatman C.E. (2016). Mechanisms, Prediction, and Prevention of ACL Injuries: Cut Risk with Three Sharpened and Validated Tools. J. Orthop. Res..

[54] Carcia C.R., Drouin J.M., Houglum P.A. (2006). The Influence of a Foot Orthotic on Lower Extremity Transverse Plane Kinematics in Collegiate Female Athletes with Pes Planus. J. Sports Sci. Med..

